# Study on Enhancement Principle and Stabilization for the Luminol-H_2_O_2_-HRP Chemiluminescence System

**DOI:** 10.1371/journal.pone.0131193

**Published:** 2015-07-08

**Authors:** Lihua Yang, Maojun Jin, Pengfei Du, Ge Chen, Chan Zhang, Jian Wang, Fen Jin, Hua Shao, Yongxin She, Shanshan Wang, Lufei Zheng, Jing Wang

**Affiliations:** Key Laboratory for Agro-Products Quality and Safety, Institute of Quality Standards & Testing Technology for Agro-Products, Chinese Academy of Agricultural Sciences, Beijing, 100081, China; University of Colorado Denver, UNITED STATES

## Abstract

A luminol-H_2_O_2_-HRP chemiluminescence system with high relative luminescent intensity (RLU) and long stabilization time was investigated. First, the comparative study on the enhancement effect of ten compounds as enhancers to the luminol-H_2_O_2_-HRP chemiluminescence system was carried out, and the results showed that 4-(imidazol-1-yl)phenol (4-IMP), 4-iodophenol (4-IOP), 4-bromophenol (4-BOP) and 4-hydroxy-4’-iodobiphenyl (HIOP) had the best performance. Based on the experiment, the four enhancers were dissolved in acetone, acetonitrile, methanol, and dimethylformamide (DMF) with various concentrations, the results indicated that 4-IMP, 4-IOP, 4-BOP and HIOP dissolved in DMF with the concentrations of 0.2%, 3.2%, 1.6% and 3.2% could get the highest RLU values. Subsequently, the influences of pH, ionic strength, HRP, 4-IMP, 4-IOP, 4-BOP, HIOP, H_2_O_2 _and luminol on the stabilization of the luminol-H_2_O_2_-HRP chemiluminescence system were studied, and we found that pH value, ionic strength, 4-IMP, 4-IOP, 4-BOP, HIOP, H_2_O_2 _and luminol have little influence on luminescent stabilization, while HRP has a great influence. In different ranges of HRP concentration, different enhancers should be selected. When the concentration is within the range of 0~6 ng/mL, 4-IMP should be selected. When the concentration of HRP ranges from 6 to 25ng/mL, 4-IOP was the best choice. And when the concentration is within the range of 25~80 ng/mL, HIOP should be selected as the enhancer. Finally, the three well-performing chemiluminescent enhanced solutions (CESs) have been further optimized according to the three enhancers (4-IMP, 4-IOP and HIOP) in their utilized HRP concentration ranges.

## Introduction

ELISA is currently one of the most widely used methods in the field of analysis and detection [1, 2]. The colorimetric method, fluorescence and chemiluminescence (CL) and other detection methods all make use of the special quality of peroxidase with enzymatic activity to label the immune reagent. Research has revealed that CL has higher sensitivity compared with other detection methods [3–5]. The chemiluminescence detection method is based on the fact that peroxidase will manifest catalytic oxidation and form luminol oxide under slightly alkaline conditions. Luminol oxide will promote the generation of the 3-amidogen-dimethyl phthalate ion in an excited state; it will emit light when it is returning to the ground state, and the maximum wavelength of the emitted light will be 425nm.

Because the catalytic ability of peroxidase is very weak when luminal oxide is formed [6], adding some compounds as enhancers to the oligomer will enhance the luminescent intensity. The enhancer will play the role of a medium during the peroxidase process [7]. The enhancer in the reaction liquid will not only have no influence on the chemical property of the end products, but it will also enhance the chemiluminescent intensity because compound I and II have a higher reactivity corresponding to the luminol. At present, most of the efficient enhancers are phenols. Many compounds have successfully been used to enhance peroxidase and catalyze luminescence [8–10].

In recent years, chemiluminescence immunoassay technology based on the HRP catalyzing Luminol-H_2_O_2_ chemiluminescence system is rapidly developing along with the development of chemiluminescence immunological technology. HRP is an essential enzyme in the field of biochemistry which has gained profound significance in detection of hydrogen peroxide or in reaction with other compounds coupled by the enzymatic reaction, contributing to the further development of the fast and sensitive CLEIA method. HRP can catalyze the reaction between a hydrogen acceptor (oxidizing agent, such as hydrogen peroxide) and hydrogen donor (chemiluminescence substrate, such as luminol). If a luminescence system using luminol as the luminescent substance doesn’t use an enhancer, then the luminescent duration and the signal of luminol will be very short. When an enhancer is added; it will enhance the luminescent intensity and prolong the luminescent time. Using different enhancers in the Luminol-H_2_O_2_-HRP system will not only improve luminescent signal intensity, but also influence luminescence kinetics [11].

Among the commonly used enhancers, there are relatively more studies on using phenol derivatives with different substituent groups as enhancers, but the defects of an insufficient lowest limitation of detection and bad repeatability still exist. 4-IOP is widely used in enhancing HRP catalyzing luminol oxidation luminescence [12, 13]. Enhanced chemiluminescence reactions (ECR) are characterized by strong luminescent intensity, stability and long duration [14]. Various phenol substitutes have been used as luminol signal enhancers, such as firefly fluorescein, 6-phenolderivatives [15], arylboronic acid derivatives, such as 4-phenylboronic acid [16], (1,2,4-triazole-1-base)phenol [17] and a series of para-position phenol derivatives [18, 19] and even more complicated analogues [20, 21].

At present, some studies have reported on using 4-IOP and 4-(1-imidazolyl) phenol (4-IMP) in the luminol-H_2_O_2_-HRP system as the chemiluminescence enhancers [9, 11]. 4-IMP has a different limit of detection and linearity range from the enhancer 4-IOP, attributed to the different 4-substituent group. The characteristics of the 4-substituent group, including an electron donor group and electron acceptor group, carrying an electric charge, an existing heteroatom and other elements, have a great influence on the O-H key dissociation of the phenol group, further influencing the stabilization of the phenoxy radical [22]. The enhancement principal of contrapuntal phenol derivatives with different substituent groups as the enhancer in the luminol-H_2_O_2_-HRP chemiluminescence system still cannot be confirmed. The para-orienting group has two functions: it will enhance the electron density in the oxygen of the phenolic hydroxyl group through the property of electron donation; and it will position the free radical electron on the para-position radical through the inductive effect of para-orientation [12]. The essential component of chemiluminescence enzyme analysis is the enhancer, which will play a decisive role in the sensitivity of detection. Hence, research and design of a new type of highly efficient and stable enhancer will become a growing trend in the future.

There have been reports that using different enhancers in the luminol-H_2_O_2_-HRP system will not only improve the intensity of the luminescent signal, but also influence the luminescence kinetics curve [12, 23]. This paper will conduct chemical dynamic testing on other factors of influence on the luminescence system, including the influences of HRP concentration and sensitization fluid, and we will do further study on luminescence stability, and select different suitable enhancers in terms of different HRP concentration ranges, so as to provide a reference for chemiluminescence analysis technology.

## Experimental Section

### Materials and Apparatus

Horseradish Peroxidase (HRP), luminol, HIOP, 4-IOP, 4-BOP, 4-IMP, 4-methoxyphenol (4-MYP), 2-iodophenol (2-IOP), 1-bromo-4-iodobenzene (4-BIB), 3-iodophenol (3-IOP), 4-phenylphenol (4-PYP), 4–4'-diiodobiphenyl (DIOP), and Trometamol (Tris) were purchased from Sigma-Aldrich (St. Louis MO, USA). Methanol, acetonitrile, acetone, DMF, N-hydroxysuccinimide (NHS), N, N'-dicyclohexyl carbodiimide (DCC), Tween 20 and hydrogen peroxide (H_2_O_2_) were supplied by Beijing Chemical Works (Beijing, China). All aqueous solutions and buffers were prepared using deionized water (resistivity > 18MΩ. cm), and all other chemicals and organic solvents were of analytical grade or better.

The white opaque 96-flat-bottomed well plates were purchased from Corning (COSTAR, NY, USA). Plates were washed with a DEM plate washer (Beijing Tuopu Analytical Instruments Co. Ltd., China). The pH of all buffer solutions was measured using a pH meter ((METTLER TOLEDO, China)). All chemiluminescent intensity measurements were performed with Multi+ Detection System with Instinct Software (Promega, WI, USA). Deionized water was purified with a MilliQ system (Waters, MA, USA).

### Preparation of CES

Based on the previous literature [19, 20, 24–27], CES has basically 6 influencing factors, including that the organic solvent will allow the enhancer to be dissolved well in the hydrofacies luminescence system; the ion intensity of the Tris-HCI buffer solution ion intensity and pH value can provide an alkaline buffer system, so as to smooth the chemiluminescence reaction; the hydrogen peroxide functions as an oxidizing agent; luminol is a commonly used HRP luminescence substrate; the enhancer can effectively enhance the chemiluminescence intensity and improve the sensitivity.

The enhancers were dissolved in organic solvents to prepare the mother solutions of the concentrations in 1000 mM. Hydrogen peroxide and luminol were prepared with Tris-HCl buffer solution (0.01 M, pH 9.0) as mother solutions with the concentrations in 200 mM and 30mM. The CESs were prepared with the Tris-HCl buffer (0.01 M, pH 9.0), including the enhancer, hydrogen peroxide and luminol which were dissolved in organic solvent.

### Chemiluminescence Detection Method

The multichannel micropipettor was used to add 50 μL HRP solvent in the 96-flat-bottomed well plates, then 150 μL CES was added separately. Finally, the multifunctional analytic detector is used to measure and record the RLU. All the experiments were carried out under ambient room temperature.

### Selection of 10 Enhancers

This study is based on previous research, and 10 compounds were chosen as enhancers, first, according to the different substitute positions, we selected 2-IOP, 3-IOP, 4-IOP to conduct comparative experiments; second, according to the para-position substituent groups, we selected 4-IOP, 4-MYP, 4-PYP, 4-BOP and 4-IMP to conduct comparisons; third, to conduct comparative study on phenol and other representative benzene compounds, we selected 4-BIB, HIOP and DIOP.

The ten enhancers were dissolved in DMF, and the CESs were prepared with each enhancer separately in a series of concentrations including 0.05 mM, 0.10 mM, 0.15 mM, 0.20 mM, 0.25 mM, 0.50 mM, 1.00 mM, 2.00 mM, 4.00 mM, 8.00 mM, and 10.00 mM. Subsequently, the enhancement effect of each enhancer in different concentrations was evaluated by the detected RLU values of each corresponding chemiluminescence system, and the RLU was measured 5 minutes after the CES was added to 96-flat-bottomed well plates.

### Selection of Organic Solvents

The tolerance of chemiluminescence to methanol, acetonitrile, acetone and DMF was evaluated in the range of 0.1%, 0.2%, 0.39%, 0.78%, 1.56%, 3.12%, 6.25%, 12.5%, 25%, 50% solvent concentration (v/v). In this case, enhancement of chemiluminescence was performed using chemiluminescent enhancer, luminol, HRP and H_2_O_2_ dissolved in 0.1 M Tris-HCl buffer (pH 8.5) containing different amounts of methanol, acetonitrile, acetone and DMF.

### Tris-HCl Buffer Effect

The influences of pH and salt concentrations of Tris-HCl buffer on enhanced chemiluminescence performance were studied using buffers of various pH values and different concentrations. First, the buffers were 100 mM Tris at different pH values (6.0, 6.5, 7.0, 7.5, 8.0, 8.5, 9.0, 9.5), which were prepared by changing the amounts of HCl, whereas the concentration of Tris remaining at 100 mM. Subsequently, the pH of all buffers was kept between 8.4 and 8.5, while different concentrations (0.002M, 0.01M, 0.05M, and 0.1M) of Tris were dissolved in the buffer. The enhancement of chemiluminescence was performed in the presence of chemiluminescent enhancer, luminol, HRP and H_2_O_2_.

### Luminol Effect

To study the effect of the luminol on enhanced chemiluminescence, different concentrations of luminol (0, 0.15 mM, 0.3 mM, 0.6 mM, 1.2 mM, 2.4 mM, 4.8 mM) were prepared in the CES.Chemiluminescence enhancement was performed together with chemiluminescent enhancer, HRP and H_2_O_2_.

### H_2_O_2_ Effect

The influences of H_2_O_2_ were investigated with various concentration including 0.13 mM, 0.25 mM, 0.5 mM, 1.0 mM, 2.0 mM, 4.0 mM, 8.0 mM of H_2_O_2_ in the CES. The chemiluminescence enhancement was performed with chemiluminescent enhancer, HRP, luminol and different concentrations of H_2_O_2_.

### HRP Effect

To investigate the influence of HRP on enhanced chemiluminescence, different concentrations of HRP (1 ng/ml, 2 ng/ml, 3 ng/ml, 4 ng/ml, 5 ng/ml, 6 ng/ml, 7 ng/ml, 8 ng/ml, 9 ng/ml, 10 ng/ml, 15 ng/ml, 20 ng/ml, 25 ng/ml, 30 ng/ml, 35 ng/ml, 40 ng/ml, 45 ng/ml, 50 ng/ml, 80 ng/ml) were prepared. The chemiluminescence enhancement was performed with chemiluminescent enhancer, luminol and H_2_O_2_ and different concentrations of HRP.

## Results and Discussion

### Selection of Enhancers and Concentration Optimization

Different enhancers will have different chemiluminescence effects on the luminol-H_2_O_2_-HRP system, so the chemiluminescence value was measured under 10 enhancer conditions.

**Fig 1:**
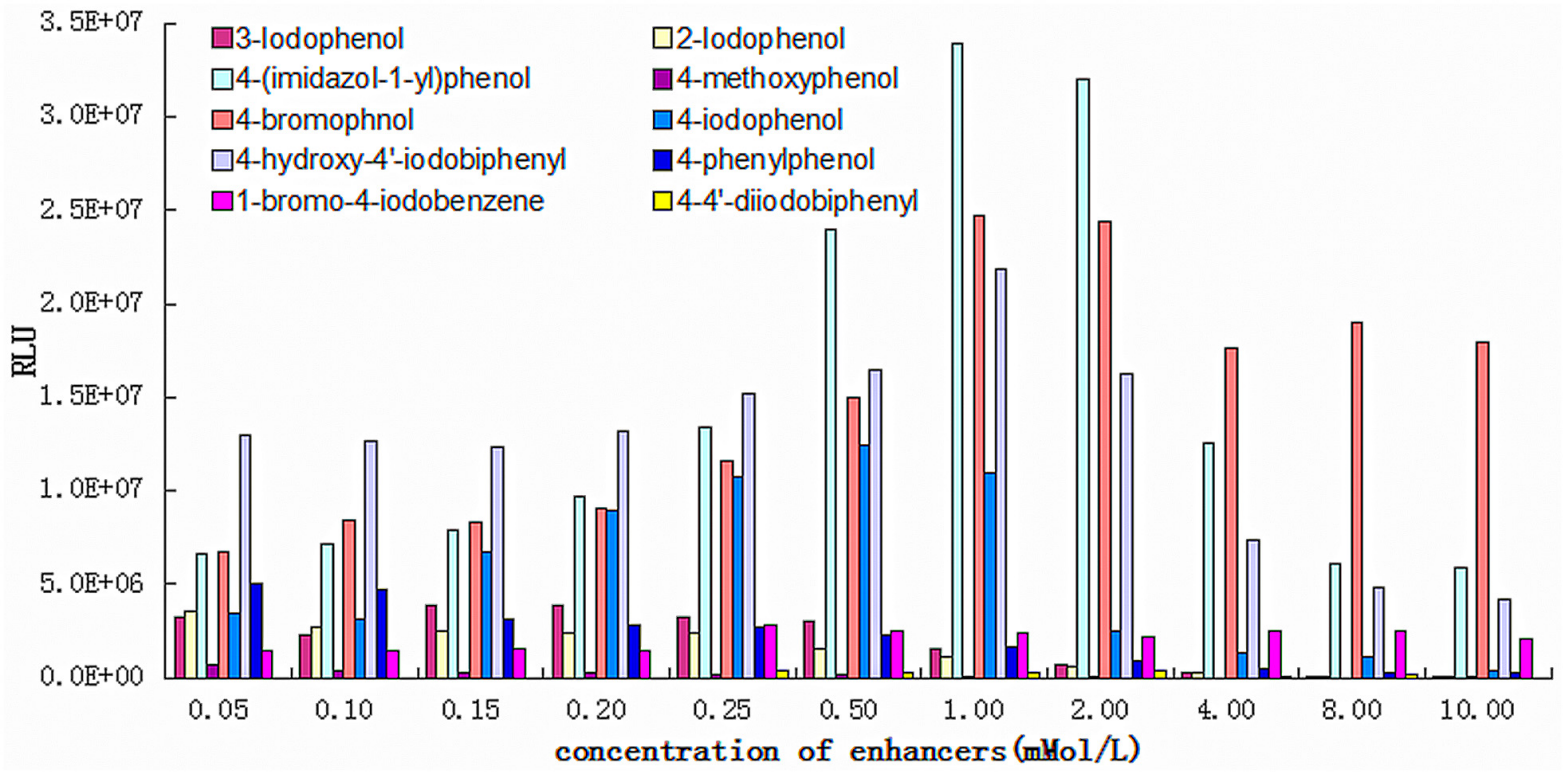
The effects of enhancers on the CL intensity on the luminol—H_2_O_2_–HRP system.

The research found that the ranking sequence of luminescence-enhancing effects for the 10 enhancers from least to greatest is: DIOP<4-MYP<4-BIB<2-IOP<3-IOP<4-PYP<4-IOP<HIOP<4-BOP<4-IMP. The luminescence-enhancing effect can be obtained with different concentrations of different enhancers, and the most excellent enhancing effect concentration can be selected, please see Fig 1. As the precise mechanism of the HRP-catalyzed chemiluminescent oxidation of luminol in the presence of a p-phenol derivative has not been 100% proved. However, phenoxyl radical generation was the main factor to affect the HRP-catalyzed chemiluminescent oxidation of luminol. It was assumed to affect the chemiluminescent intensity via the electron transfer between radicals and luminol. Based on previous study with different 4-substituent phenols, we thought that the electronic properties (i.e. extent of resonance effect) of the substituents play the critical role on radical stabilization and therefore on chemiluminescent intensity enhancement. As 4-substituent of 4-IMP including an aromatic ring and with heteroatoms of nitrogen, it could provide a resonance stabilization of the phenoxyl radicals through π-delocalization. Also, electron donating groups have a similar effect (reduction) on O-H bond dissociation energy, and therefore stabilize phenoxyl radicals [19]. So we thought it was the reason why 4-IMP could get the best enhancement effect.

From Fig 1 we can see that the CESs prepared by 2-IOP, 3-IOP, 4-MYP, 4-BIB and 4-PYP generally have a low enhancing effect; the RLU basically reaches 10^5^~10^6^ order of magnitude. The first two may result from the iodine not being on the para-position, which has inferior luminescence-enhancing effects compared with 4-phenol. The latter three may result from poor solubility, and a white precipitate which may be generated when adding to the hydrofacies sensitization fluid system. These are not suitable to be used as enhancers.

The results indicated (see Fig 1 and S1 Text) that when the concentration of 4-IMP is 1mM, the luminescence-enhancing effect is best. The luminescence value decreases as the concentration of 4-IMP increases, which may be due to its solubility in the hydrofacies system being reduced, resulting in decreased enhancing effect along with the increased concentration of 4-IMP. The luminescence value is increased as the concentration of 4-BOP increases. When the concentration reaches 1mM, the luminescence-enhancing effect is best, then it begins to decrease. When the concentration of 4-IOP is 0.5mM, the luminescence-enhancing effect is best, and the luminescence value decreases along with the increase of concentration. When the concentration of HIOP reaches 1mM, the luminescence-enhancing effect is best, and when its concentration is increasing or decreasing, the luminescence intensity will decrease. The maximum luminescence value of the four enhancers, 4-IMP, 4-BOP, 4-IOP and HIOP will reach the 10^7^ order of magnitude, and the optimal values of chemiluminescence-enhancing effects are respectively 1mM, 1mM, 0.5mM and 1mM (Please see Fig 1)

The experimental results show that phenol compounds with different substituent positions will have different enhancing effects. An enhancing para-position substituent will have a better luminescence effect compared with other positions; the imidazole group on the para-position will have better luminescence-enhancing effects compared with the halogenate group, while the halogenate group has better luminescence effects than the methoxy group and phenyl group. The phenol compounds with hydroxyl will have better luminescence-enhancing effects than 4-bromine iodobenzene, 4–4 diphenylene iodonium and 4-hydroxy-4-iodine biphenyl compounds. Four types of enhancers with better enhancing effects can therefore be chosen, such as 4-(1-radical-imidazole), phenol, 4-bromophenol, 4-iodophenol and 4-hydroxy-4-iodine biphenyl. Based on the results, 1.0 mM 4-IMP was selected in the sections of “The Influence of pH and salt concentrations of Tris-HCl buffer”, “H_2_O_2_ Influence” and “Luminol Influence”. Meanwhile, 1.0 mM 4-IMP, 1.0 mM 4-BOP, 1.0 mM HIOP and 0.5 mM 4-IOP were used in the section of “HRP Influence”.

### The Influence of Organic Solvent on the Luminescence System

As the enhancers are generally difficult to dissolve in the hydrofacies luminescence system, they need to be pre-dissolved with organic solvent. This paper has chosen four commonly-used solvents, like methyl alcohol, acetonitrile, acetone and DMF, which were used to dissolve the 10 enhancers separately. We found that 4-IOP, 4-BOP, 2-IOP, 3-IOP, 4-PYP and 4-MYP could be dissolved in methyl alcohol, acetonitrile, acetone and DMF; 4-IMP can only be dissolved in DMF; 4-BIB and HIOP could be dissolved in acetone and DMF; and DIOP can’t be dissolved in the four organic solvents. The results show that DMF can dissolve most of the enhancers and is an excellent solvent compared to the other three.

Organic solvents should be pre-dissolved in the luminol—H_2_O_2_–HRP-Enhancer luminescence reaction system due to the poor solubility of some enhancers in hydrofacies luminescence systems. This paper has aimed to conduct a comparison among the four high quality organic solvents DMF, carbinol, acetonitrile and acetone without influence on the chemiluminescence system, so as to choose the best organic solvent.

Based on section 2.1, this study has selected 4-IMP, 4-BOP, 4-IOP and HIOP as the enhancers with good enhancing effect, and we will study the influence of the four organic solvents methyl alcohol, acetonitrile, acetone, DMF on the enhancing effect of four chemiluminescence systems: luminol-H_2_O_2_-HRP-HIOP, luminol-H_2_O_2_-HRP-4-IOP, luminol-H_2_O_2_-HRP-4-BIP and luminol-H_2_O_2_-HRP-4-IMP.

**Fig 2:**
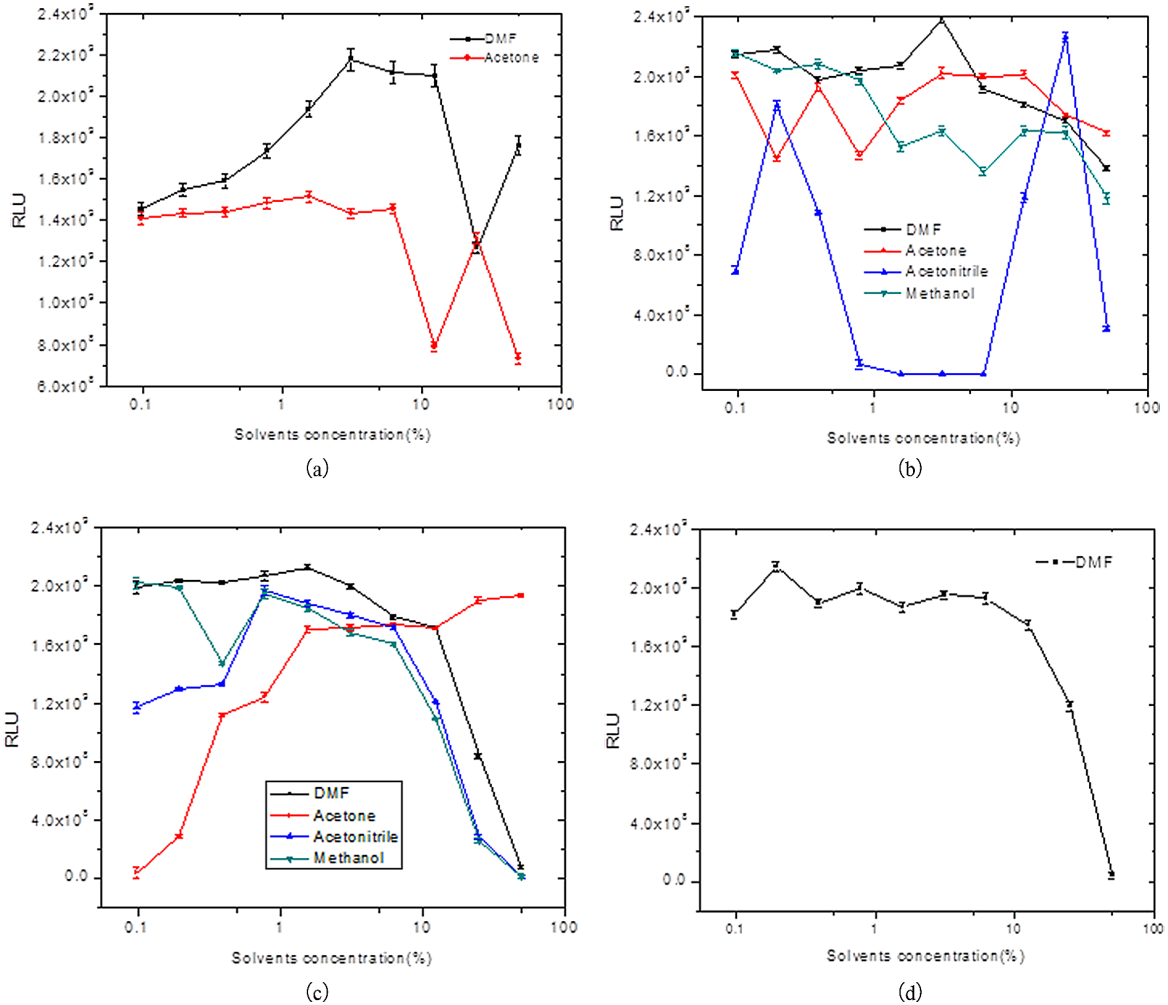
The effect of organic solvent concentration on the CL intensity. a) Luminol-H_2_O_2_-HRP-HIOP, b) Luminol-H_2_O_2_-HRP-4-IOP, c) Luminol-H_2_O_2_-HRP-4-BOP, d) Luminol-H_2_O_2_-HRP-4-IMP.

HIOP dissolved by DMF has better luminescence-enhancing effects compared with HIOP dissolved by acetone, with an enhanced luminescence value reaching 10^9^. The luminescence value will increase along with the increase of DMF content (see Fig 2a and S2 Text), which may be because HIOP is difficult to dissolve in water, and will separate out a white precipitate in a hydrofacies luminescence system. As Fig 2b and S3 Text demonstrate, the order of magnitude of 4-IOP enhanced luminescence reaches 10^9^; the enhancement effects of DMF are relatively good, while the enhancement effects of acetonitrile are the least. The influence of organic solvent in terms of luminescence-enhancing effects is very small. As Fig 2c and S4 Text show, when the content of DMF in the luminescence system reaches 1.6%, the dissolved 4-BOP enhancement effect is best and luminescence is at the maximum value. Along with the increase of acetone content, the enhanced luminescence of 4-BOP is increased too, with its luminescence value reaching 10^9^. When using DMF to dissolve 4-IMP, the enhanced luminescence will reach 10^9^; when the content of DMF is greater than 10%, the effect decreases (see Fig 2d and S5 Text). As in summary, if the concentration of organic solvents in the buffer was too low, the enhancer may be precipitated in the buffer system and the RLU will at the low level. And if the concentration of organic solvents in the buffer was too high, it could affect the activity of the HRP and decrease the RLU. So we thought the combination of two factors mentioned above was the key factor how the concentrations of organic solvents affect the RLU of the chemiluminscent system.

HIOP, 4-IOP, 4-BOP and 4-IMP can all be dissolved in DMF, and when the content of DMF reaches 3.2%, 3.2%, 1.6% and 0.20% respectively, the four chemiluminescence systems obtain the optimized enhanced effect, as seen in Fig 2.

The experiment results have shown that the chemiluminescence value will vary as the organic solvents vary. In terms of the several enhancers with better enhancing effect, using enhancers dissolved by DMF will enhance the chemiluminescence value with better effect than those dissolved by methyl alcohol, acetonitrile and acetone. It may result from the phenol compounds dissolved by DMF easily dissolving in the hydrofacies luminescence system, while the enhancers dissolved by methyl alcohol, acetonitrile and acetone will separate out a lot of solids when added to the hydrofacies sensitization system, resulting in weak effect.

The experimental results have also shown that the chemiluminescence value will vary as the content of organic solvents varies. Generally, the chemiluminescence value will drop as the content of organic solvents drops. However, the enhancing effect of 4-phenylphenol and 4-hydroxy-4-iodine biphenyl will increase as the percentage of DMF content increases, and the chemiluminescence value will be increased. This phenomenon could be explained by the fact that 4-phenylphenol and 4-hydroxy-4-iodine biphenyl will have more powerful solubility in DMF and water-mixed sensitization fluids. It may also be because there is a kind of ambiguous promotion effect existing in DMF.

### Chemiluminescence Kinetic Curve of luminol-H_2_O_2_-HRP-Enhancer System

With Chemiluminescence Kinetics as our research method, we have made a systematic study on the influence of the pH and salt concentrations of Tris-HCl buffer, hydrogen peroxide, luminol and enhancers in the Chemiluminescence reaction system on luminol-H_2_O_2_-HRP-Enhancer luminescence system stability. Constant Time (CT) is defined as the duration of time that the rate of change of the luminescence value is less than 1%. The larger the CT value, the longer the observed stable chemiluminescence time, which better facilitates the stability of detection methods such as CLEIA. So we take the CT as the indicator to evaluate the stability of the chemiluminescent system. The pH value, the influence of pH value, salt concentration, hydrogen peroxide and luminol on curve variation in Chemiluminescence reaction kinetics is very small; i.e. under different concentrations, the chemiluminescence value will vary, while the curve variation principles of Chemiluminescence reaction kinetics are basically consistent.

#### The Influence of pH and salt concentrations of Tris-HCl buffer

**Fig 3:**
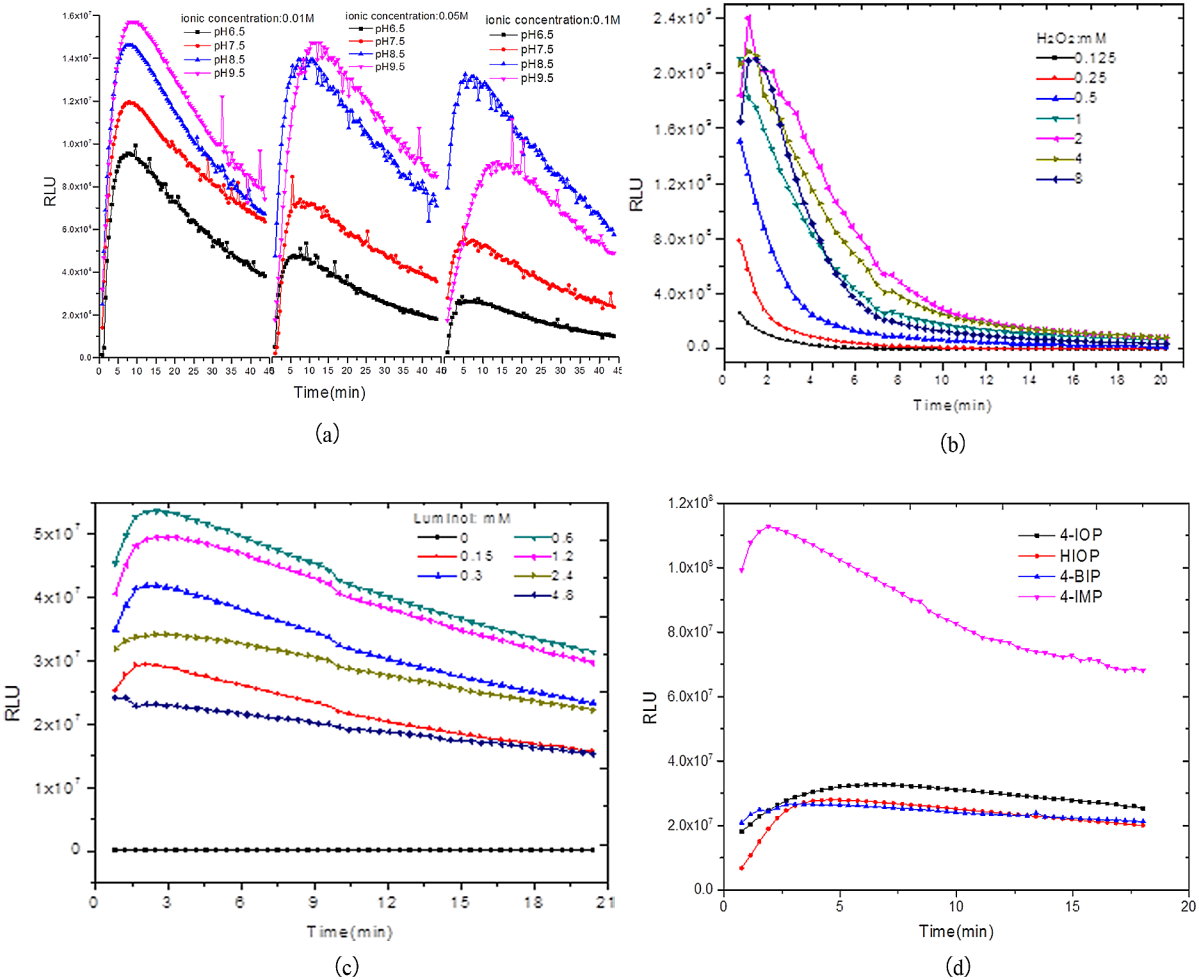
The CL kinetic curves for the luminol-H_2_O_2_-HRP-enhancer system. a) pH and Ionic concentration, b)H_2_O_2_, c)Luminol, d)Enhancer.

The CL intensity in the chemiluminescence kinetic curve and the stability of the CL-kinetic decline along with the increase of salt concentration, while CL intensity increases along with the increase of pH value. When the salt concentration of the Tris-HCI buffer is 0.01M and the pH value is 9.5, the CL intensity value is greatest (see Fig 3(a) and S6 Text).

#### H_2_O_2_ Influence

When H_2_O_2_ is under low concentration, the CL intensity will increase along with the increase of H_2_O_2_; when H_2_O_2_ is 2mM, the CL intensity reaches its maximum value, and when H_2_O_2_ is higher than 2mM, the CL-RLU decreases instead. At different concentrations of H_2_O_2_, the CL-kinetic variation principle is basically uniform: all decrease quickly at first, then maintain a slow decrease, while the influence of H_2_O_2_ concentration on the stability of the chemiluminescent system is very small (Please see Fig 3(b) and S7 Text).

#### Luminol Influence

When luminol is at a low concentration, the CL-RLU will increase along with the increase of luminol; when luminol is under 0.6 mM, the CL-RLU reaches its maximum value, while when luminol is higher than 0.6 mM, the CL-RLU decreases instead. At different concentrations of luminol, the variation principle of CL-kinetic is basically uniform: all increase at first, then drop, while the influence of luminol concentration on the stability of the chemiluminescent system is very small (Please see Fig 3(c) and S8 Text).

#### Enhancer Influence

In the luminescence system, the CL-RLU dramatically varies when different enhancers are added, and when the same enhancer is added, the CL-kinetic variation principle is basically uniform along with the changes in its concentration (please see S9 Text). All increase at first, then maintain stability, then drop slowly. The CL-kinetic is very high for different enhancers under optimized enhancer concentrations (please see Fig 3(d)), this may result from the enhanced luminescence effect of different enhancers.

#### HRP Influence

The fact that luminescence strength will increase along with the increase of HRP concentration has been widely accepted. This research carried out observations on the four enhancers HIOP, 4-IOP, 4-BOP and 4-IMP under optimized concentrations with HRP in different concentrations, and investigated the influence of HRP concentration on CL intensity and stability.

Experimental results (please see Table 1) have shown that the maximum luminescence RLU(max) of the four enhancers all progressively increase as HRP concentration increases, the time to reach the maximum value, i.e. Time (RUL max) will be shortened, and the variation trend is basically uniform. For example, 4-IOPwhen used as the enhancer, has a larger CT value when the concentration of HRP is under 10, 15, 20 and 25ng/ml, which shows that the chemiluminescence strength will have more stability; while when the concentration of HRP concentration is under 5 and 50 ng/ml, the CT value is less than 40s, and the stability of chemiluminescence is poor. We can ascertain from this that different substances should be chosen as enhancers for different HRP concentration ranges.

**Table 1:** The effect of HRP and Enhancers on CL.

Enhancer	HRP concentration	5ng/mL	10ng/mL	15ng/mL	20ng/mL	25ng/mL	50ng/mL
4-HIOP	RLU(max)	33,3639	655,3980	1508,9000	2330,8600	2796,3300	7620,8000
	Time(RLU max)(s)	1160	640	420	280	220	140
	Constant Time(s)	instability	instability	360 to 420 (60)	240 to 360 (120)	200 to 280 (80)	100 to 180 (80)
4-IOP	RLU(max)	365,1100	1433,3000	2282,5000	3266,2600	3437,5300	9534,4200
	Time(RLU max)(s)	825	725	575	400	475	175
	Constant Time(s)	instability	460 to 700 (240)	400 to 560 (160)	280 to 380 (100)	320 to 440 (120)	140 to 180 (40)
4-BIP	RLU(max)	129,4880	586,1040	1791,4200	2520,2400	2557,5500	8054,8700
	Time(RLU max)(s)	480	400	280	160	140	60
	Constant Time(s)	460 to 480 (20)	instability	200 to 360 (160)	180 to 240 (60)	80 to 240 (160)	60 to 80 (20)
4-IMP	RLU(max)	2193,4200	4314,0400	7592,8400	11325,4000	11281,3000	44702,3000
	Time(RLU max)(s)	220	160	180	200	80	40
	Constant Time(s)	200 to 300 (100)	120 to 200 (80)	80 to 140 (60)	60 to 100 (40)	80 to 100 (20)	20 (20)

**Fig 4:**
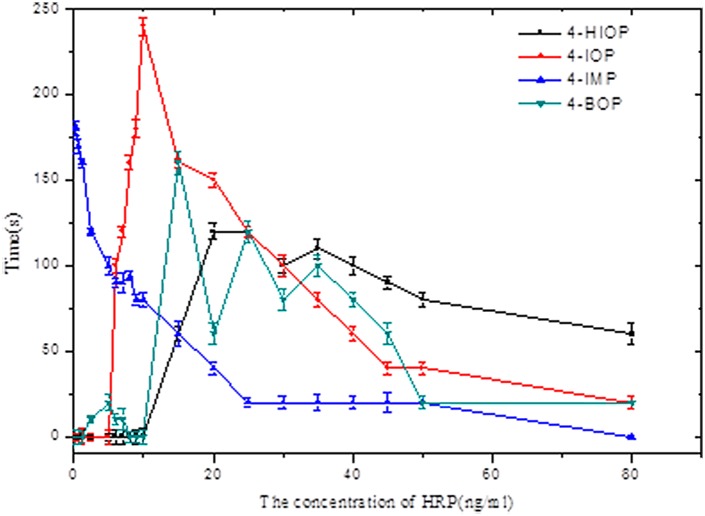
The application range of HRP concentration within the three enhancers and their respective required stability time for chemiluminescence.

In order to obtain a tighter HRP concentration range, this study will provide a further discussion of different HRP concentrations. Taking the HRP concentration as the abscissa and the 60s and above CT value as the ordinate, the relation curve of HRP and stability time can be obtained, see Fig 4. This study shows that when the HRP concentration range is from 0~6ng/mL using 4-IMP, the CT value will be the largest, and the longest stable time will be obtained; when the HRP concentration range is from 6 to 25 ng/mL, 4-IOP should be chosen, as the luminescence will be more stable; and when the HRP concentration range is from 25 to 80 ng/mL, it is better to use HIOP as the enhancer.

The experiment results have shown that the influence of Tris-HCl buffer, H_2_O_2_, Luminol and enhancer on the chemiluminescence curve is very small, while HRP concentration will have a powerful influence on the system luminescence curve. Both too low and too high luminescence strengths will result in poor stability of luminescence, so that suitable enhancers should be chosen for different HRP concentration ranges to stabilize its luminescence.

In the luminol-H_2_O_2_-HRP chemiluminescent system, HRP was the catalyst. In different concentrations of HRP, the catalytic effect to the chemiluminescent system differed a lot. We surmised the concentrations of HRP would affect the reaction rate of the HRP-catalyzed CL oxidation of luminol in the chemiluminescent system, and subsequently affect the duration time of chemiluminescence. So in the paper, with the function of suitable enhancer, HRP has a great influence on luminescence stability.

### Optimization of CES Conditions

Based on the above research, the three well-performing enhanced chemiluminescent solutions, including pH and salt concentrations of the Tris-HCl buffer, H_2_O_2_ and Luminol in the chemiluminescence reaction luminol—H_2_O_2_–HRP-Enhancer system, can be further optimized according to the three enhancers (4-IMP, 4-IOP and HIOP) in their utilized HRP concentration ranges.

**Fig 5:**
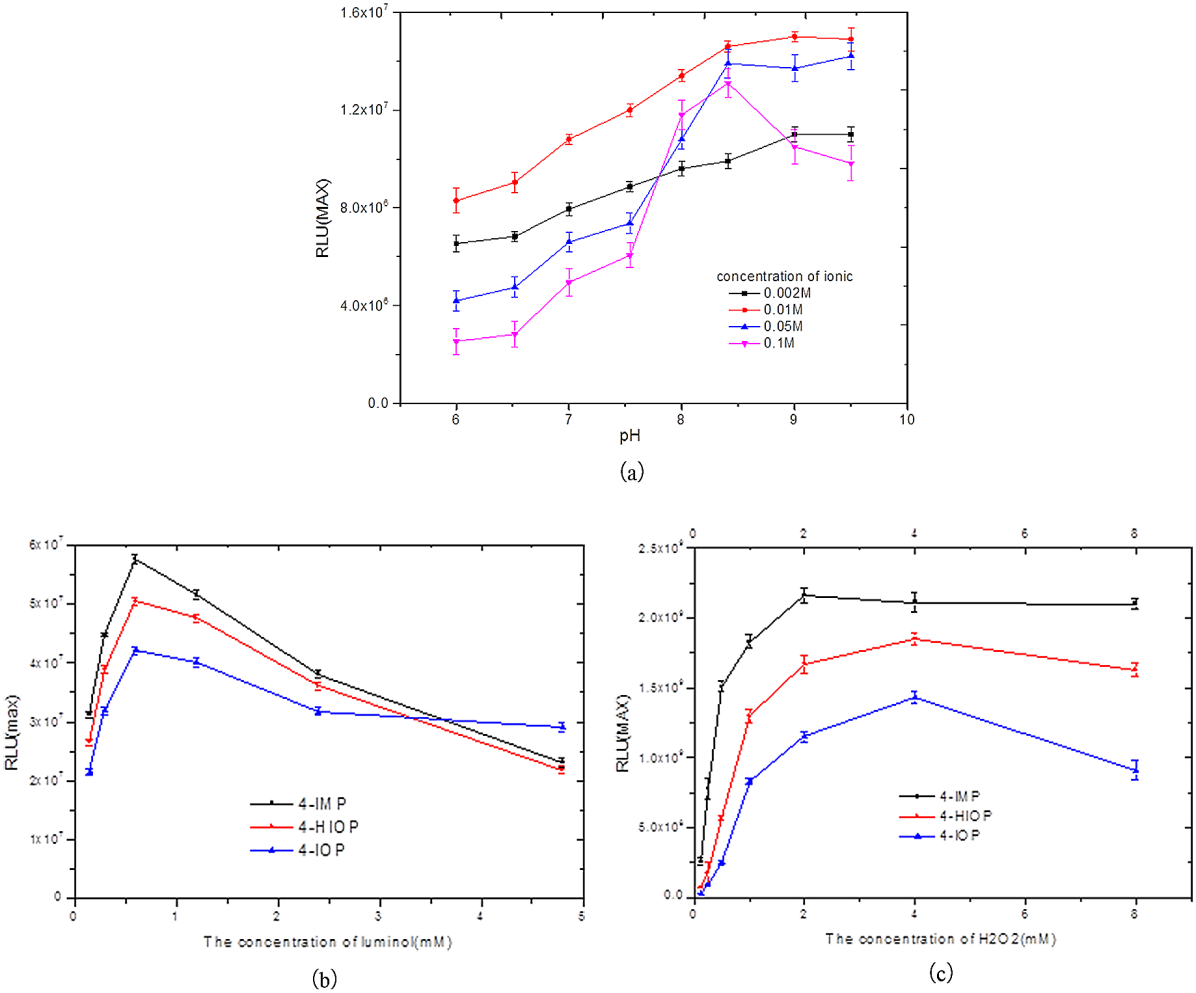
Optimization of concentration of ionic and pH value (a), H_2_O_2_ (b), Luminol (c).

In the 4-IMP enhancing system, the system luminescence will be highest when the salt concentration is under 0.01M; as the pH value rises, the luminescence strength will increase. When the pH value reaches 9.0 it tends to smooth out (please see Fig 5(a)). Therefore, Tris-HCI buffer fluid (0.01M, pH9.0) provides the best chemiluminescence reaction conditions, and the enhancing system of 4-IOP and HIOP will show the same result as 4-IMP; it will present the maximum luminescence value under the condition of Tris-HCI buffer fluid (0.01M, pH9.0). Hence, the optimized Tris-HCI buffer condition for the three enhancer system is 0.01M as the salt concentration and 9.0 as the pH value.

When the concentration of luminol is increased, RLU (max) will increase first and then decrease; the optimized luminol concentration for all three enhancers 4-IMP, 4-IOP and HIOP is 0.63mM (please see Fig 5(b)). RLU (max) will increase first and then decrease along with the increase of H_2_O_2_ concentration. The optimized H_2_O_2_ concentrations for the three enhancers 4-IMP, 4-IOP and HIOP are 2mM, 4mM and 4mM, please see Fig 5(c).

To sum up, the optimized formulas for the three enhancing sensitization fluids will be obtained within a limited HRP range in the luminol—H_2_O_2_–HRP-Enhancer luminescence reaction system (see Table 2).

**Table 2:** Three formulations of the Luminol-H_2_O_2_-HRP-enhancer CL system.

	4-IMP	4-IOP	HIOP
DMF (%)	0.20	3.2	3.2
Luminol (mM)	0.63	0.63	0.63
H_2_O_2_ (mM)	2	4	4
Enhancer (mM)	1	0.5	1
pH and Ionic (M)	9.0, 0.01	9.0, 0.01	9.0, 0.01
HRP (ng/ml)	0~6	6~25	25~80

## Conclusions

We have selected three enhancers (4-IMP, 4-HIOP and 4-IOP) with the best enhancing effect in 10 compounds. Comparing the four excellent organic solvents DMF, methyl alcohol, acetonitrile and acetone, we found that DMF will have better dissolution in a chemiluminescence system and has a certain promotion effect. The study also found that the influence of H_2_O_2_, Luminol and enhancers on luminescence stability is very small, while HRP has a great influence on luminescence stability. Under different HRP concentration ranges, different enhancers should be chosen: when the HRP concentration ranges from 0~6 ng/mL with 0.20% DMF, 2 mM H_2_O_2_ and 0.63 mM Luminol, 1 mM 4-IMP should be chosen, when the HRP concentration ranges from 6–25 ng/mL with 3.2% DMF, 4 mM H_2_O_2_ and 0.63 mM Luminol, 0.25 mM 4-IOP should be chosen; and when the HRP concentration ranges from 25–80 ng/mL with 3.2% DMF, 4 mM H_2_O_2_ and 0.63 mM Luminol, 1 mM HIOP should be chosen.

## Supporting Information

S1 Text The RLU values of ten enhancers in different concentrations. (XLS)

S2 Text The RLU values of HIOP dissolved in acetone and DMF at different concentrations. (XLS)

S3 Text The RLU values of 4-IOP dissolved in methanol, acetone, DMF and acetonitrile at different concentrations. (XLS)

S4 Text The RLU values of 4-BOP dissolved in methanol, acetone, DMF and acetonitrile at different concentrations. (XLS)

S5 Text The RLU values of 4-IMP dissolved in DMF at different concentrations. (XLS)

S6 Text The effect of pH and ionic concentration of the tris-HCl buffer to CL kinetic curves. (XLS)

S7 Text The effect of H_2_O_2_ concentrations to CL kinetic curves. (XLS)

S8 Text The effect of luminol concentrations to CL kinetic curves. (XLS)

S9 Text The effect of 4 enhancers concentrations to CL kinetic curves. (XLS)
